# RNA-sequencing-based detection of human viral pathogens in cerebrospinal fluid and serum samples from children with meningitis and encephalitis

**DOI:** 10.1099/mgen.0.001079

**Published:** 2023-08-02

**Authors:** Guohao Fan, Sai Li, Fengyu Tian, Longgui Yang, Suwu Yi, Sitian Chen, Chengyi Li, Ruiqing Zhang, Xiaozhou He, Xuejun Ma

**Affiliations:** ^1^​ National Institute for Viral Disease Control and Prevention, Chinese Center for Disease Control and Prevention (China CDC), Beijing 102206, PR China; ^2^​ The Third People’s Hospital of Shenzhen, Shenzheng 518112, PR China; ^3^​ Hunan Children’s Hospital, Changsha, Hunan, 410001, PR China; ^4^​ Graduate School, Hebei Medical University, Shijiazhuang 050031, PR China

**Keywords:** NGS, Virome, CSF, Serum, Meningitis, Encephalitis

## Abstract

Encephalitis and meningitis are notable global public health concerns, especially among infants or children. Metagenomic next-generation sequencing (mNGS) has greatly advanced our understanding of the viruses responsible for these diseases. However, the detection rate of the aetiology remains low. We conducted RNA sequencing and virome analysis on cerebrospinal fluid (CSF) and serum samples commonly used in the clinical diagnosis to detect viral pathogens. In total, 226 paired CSF and serum samples from 113 children with encephalitis and meningitis were enrolled. The results showed that the diversity of viruses was higher in CSF, with a total of 12 viral taxa detected, including one case each of herpesvirus, coronavirus and enterovirus, and six cases of adenovirus related to human diseases. In contrast, the *Anelloviridae* was the most abundant viral family detected in serum, and only a few samples contained human viral pathogens, including one case of enterovirus and two cases of adenovirus. The detection rate for human viral pathogens increases to 10.6 %(12/113) when both types of samples are used simultaneously, compared to CSF along 7.9 % (9/113) or serum alone 2.6 % (3/113). However, we did not detect these viruses simultaneously in paired samples from the same case. These results suggest that CSF samples still have irreplaceable advantages for using mNGS to detect viruses in patients with meningitis and encephalitis, and serum can supplement to improve the detection rate of viral encephalitis and meningitis. The findings of this study could help improve the etiological diagnosis, clinical management and prognosis of patients with meningitis and encephalitis in children.

## Data Summary

The datasets generated and analysed in this study are available in this published article (and its Supplementary Information Files, available in the online version of this article). Raw sequence reads from Illumina Novaseq were deposited into NCBI BioProject under accession number PRJNA963158 (https://www.ncbi.nlm.nih.gov/bioproject/PRJNA963158).

**Impact Statement**

Encephalitis and meningitis pose significant public concerns worldwide, particularly in infants or children. However, a large percentage 40–60% of these cases have an unknown cause. The use of metagenomic next-generation sequencing (mNGS) has helped to shed light on the viruses responsible for these conditions and led to more accurate diagnosis of their etiology. This study focused on 113 children with encephalitis and meningitis, and used mNGS to examine the viral profiles present in paired specimens of cerebrospinal fluid (CSF) and serum. The results suggest that CSF samples still have irreplaceable advantages for using mNGS to detect viruses in patients with meningitis and encephalitis, and serum can supplement to improve the detection rate of viral encephalitis and meningitis. The findings can benefit the etiological diagnosis, clinical management and prognosis of meningitis and encephalitis in children.

## Introduction

Meningitis and encephalitis are major causes of disorders in the central nervous system, particularly among infants or children, which result in high rates of morbidity and mortality [1, 2]. The conventional diagnostic approach is often insufficient and involves costly pathogen-specific diagnostics, serial testing of cerebrospinal fluid (CSF), and sometimes invasive surgical procedures. Despite the significant diagnostic efforts, 40–60 % of patients with meningitis or encephalopathy have no clear aetiology, posing challenges for targeted treatment, accurate prognostic information, and outbreak prevention [3–7].

Recent research suggests that viruses, such as enteroviruses, herpesviruses or arboviruses, may be a major factor in the development of these diseases [8]. Metagenomic next-generation sequencing (mNGS) is a powerful tool for detecting both known and novel viral agents that may have evaded traditional diagnostic methods. It enables the unbiased detection of a wide range of viral pathogens in a single test, which can be computational classified to identify potential pathogens [1, 9–12]. The use of the mNGS approach has been successful in revealing the entire virome and microbiome of various species, including the identification of pathogenic agents from CSF and serum of patients with encephalitis or meningitis [13–18]. This diagnostic approach can facilitate earlier and more targeted treatment of neuroinvasive infections, identify emerging infections and disease phenotypes, and expedite the detection and treatment of non-infectious causes. Overall, mNGS has the potential to revolutionize the diagnosis and management of these neurological conditions.

The diagnosis of encephalitis cases typically involves the collection of CSF and serum samples [14, 19, 20]. However, there is still a lack of comprehensive studies comparing the ability to detected viral infections in CSF and serum using mNGS methods. Thus, this study aimed to gain a better understanding of the virome associated with encephalitis and meningitis by analysing paired samples of CSF and serum from hospitalized children in Hunan Children’s Hospital. RNA sequencing and virome analysis tools were utilized to analyse the samples and identify viral pathogens, with a particular focus on comparing the human viral pathogens in CSF and serum. The results of this study have the potential to facilitate earlier and more targeted treatments for neuroinvasive infections, as well as the identification of emerging infections and disease prototypes.

## Methods

### Study design and sample collection

From June to September 2020, paired CSF and serum samples were collected from children who were hospitalized at Hunan Children’s Hospital (Hunan province, China). All participating children were diagnosed by the attending physician as encephalitis or meningitis patients or suspected patients according to clinical diagnostic criteria [8, 21, 22]. Prior to admission, the children had undergone routine clinical testing, including serologic testing for IgG/IgM antibodies for common viruses in children such as adenovirus, HSV, and CMV, which all yielded negative results. Additionally, nucleic acid testing confirmed that they were all negative for SARS-CoV-2. None of the patients were receiving antibacterial and antiviral therapy at the time of sampling. All samples were collected using standard medical protocols by qualified healthcare personnel. Samples were partially used for clinical diagnosis, while the remaining portions were used in this study. Furthermore, non-identifiable information such as age, gender and other relevant clinical data were obtained from the patients' medical records for subsequent analysis. The sampling and experimental procedures of this study were reviewed by the Ethics Committee of Hunan Children’s Hospital (approval number: SQ2018-32).

### Viral nucleic acid extraction, library construction and next-generation sequencing

The total nucleic acid, which includes both DNA and RNA, was extracted from the samples and negative control using the MagMAX-96 Viral RNA Isolation Kit following the manufacturer’s protocol. The extracted nucleic acids were stored at −80 °C for subsequent NGS library construction. To minimize the risk of the environment contamination, nucleic acid extraction and libraries' construction were conducted in isolated workspaces, and rigorous disinfection measures were implemented. In addition, five blank controls were included, in which only RNase-free water was used for nucleic acid extraction and library construction.

Prior to library preparation, we conducted a reverse transcription reaction (SuperScript IV reverse transcriptase, ThermoFisher) and second-strand synthesis (DNA Polymerase I, NEB) of RNA to generate cDNA. It was then subjected to multiplex displacement amplification (MDA, REPLI-g Advanced DNA Single Cell Kit, Qiagen), which is designed for whole-genome amplification from low-input DNA samples. The final product concentrations were measured using the Qubit 3.0 Flurometer (Qubit dsDNA Assay Kit, Life Technologies) to ensure that there was enough material for library construction.

Sequencing libraries were constructed using the Nextera XT DNA Sample Preparation Kit (Illumina) according to the manufacturer’s instructions. Briefly, DNA samples were fragmented, followed by ligated to connectors, and then subjected to PCR amplification. The PCR products were then purified and quantified. Sequencing was performed by Novogene (China) on the Novaseq platform (Illumina) with a length of 150 bp and paired-end sequencing.

### Bioinformatics analyses of viral metagenomic sequencing

The bioinformation analysis pipeline for the virome in this study was based on a previous protocol [23], and involved several steps. First, quality control was performed on the raw data, which was then aligned to the human reference genome (GRCh38/hg38) to determine the percentage of host background. Host genome reads were then removed, and any reads belonging to known cellular organisms (bacteria, archaea and eukaryotes) were excluded [24]. To identify viruses, the remaining viral-related reads were aligned to the NCBI Virus nucleotide and protein databases (NCBI Virus) using blastn and blastx tools, respectively. Taxonomies with aligned reads with optimal blast scores were resolved using the MEGAN6 Metagenome Analyzer (v. 6.21.10) [25]. Finally, the results of the comparison of viral reads and the results of the comparison of viral contigs were combined to generate the final results.

The criteria for considering a virus positive in a sample are that (1) its abundance should be greater than two reads per million (RPM), (2) the number of reads for that virus should represent more than 1 % of the total number of viral reads, and (3) at least one assembly fragment was able to accurately align (using a threshold of 75 % for nucleotide or amino acid sequence identity) to the corresponding virus. The calculation method of RPM is the number of reads annotated as virus divided by the total number of reads in the sample after removing the host reads, multiplied by a million. Furthermore, PCR amplification and Sanger sequencing validation on the sequences identified by metagenomic analysis as viral were performed to rule out potential sequencing or analysis errors. Statistical analysis was performed using GraphPad software (v.9.0.0, GraphPad Software, San Diego, CA, USA, www.graphpad.com), with a significance level threshold of 0.05. Figures and charts were created using GraphPad, MEGAN6 and HiPlot (https://hiplot.com.cn/).

## Results

### Baseline characteristics and clinical symptoms of participants

**Fig. 1.**
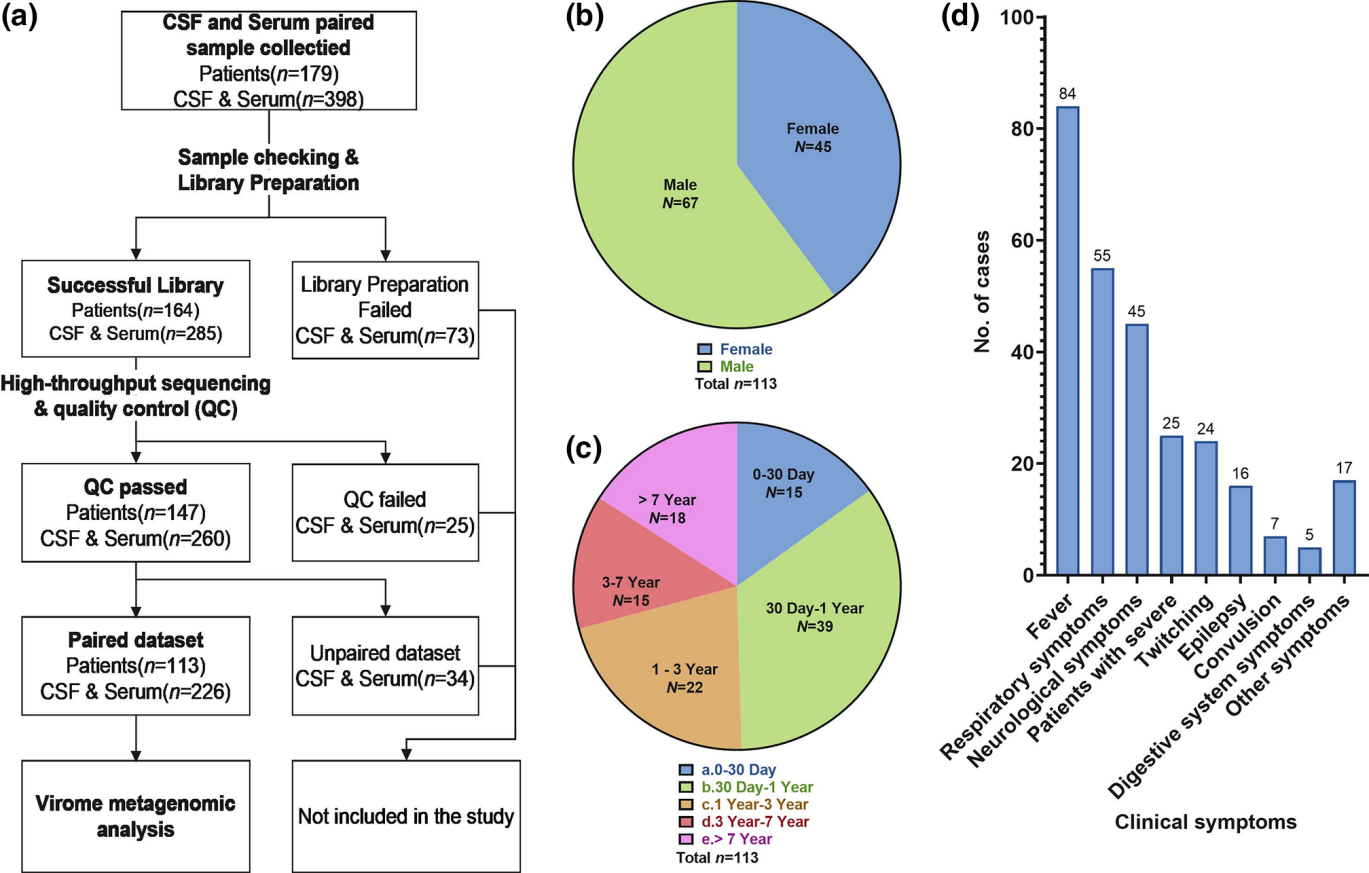
Study workflow, basic characteristics and clinical symptoms of subjects. (a) The overall workflow of this study. Figures (b), (c), and (d), respectively, show the distribution of gender, age, and clinical symptoms of the subjects.

The hospitalized paediatric patients were initially screened from July to September 2020 and identified 179 patients to be included in this study. After quality control and excluding samples with low quantity, poor nucleic acid quality, or failed sequencing library construction, 226 samples (113 CSF and 113 serum) from 113 patients were finally included in this study (Fig. 1a). Of the 113 participants, 67 were male and 45 were female (Fig. 1b), ranging in age from 1 day to 15 years. We divided them into five groups: group A (0–30 days, *n*=15), group B (30 days to 1 year, *n*=39), group C (1–3 years, *n*=22), group D (3–7 years, *n*=15), and group E (over 7 years, *n*=18) (Fig. 1c). The main clinical symptom among patients was fever (*n*=84), followed by respiratory symptoms (including upper respiratory tract infection and pneumonia, *n*=55), neurological symptoms (clear symptoms of encephalitis or meningitis, *n*=45), severe cases (patients with severe symptoms admitted to ICU, *n*=25), seizures (*n*=24), epilepsy (*n*=16), convulsions (*n*=7) and digestive system symptoms (including vomiting and diarrhoea, *n*=5). Patients with less than five cases with a single symptom were grouped as ‘other symptoms’ (*n*=17), which included symptoms such as headache, bleeding, rash, movement disorders, or altered mental status (Fig. 1d).

### Metagenomic virus taxonomic and comparison between CSF and serum

**Fig. 2.**
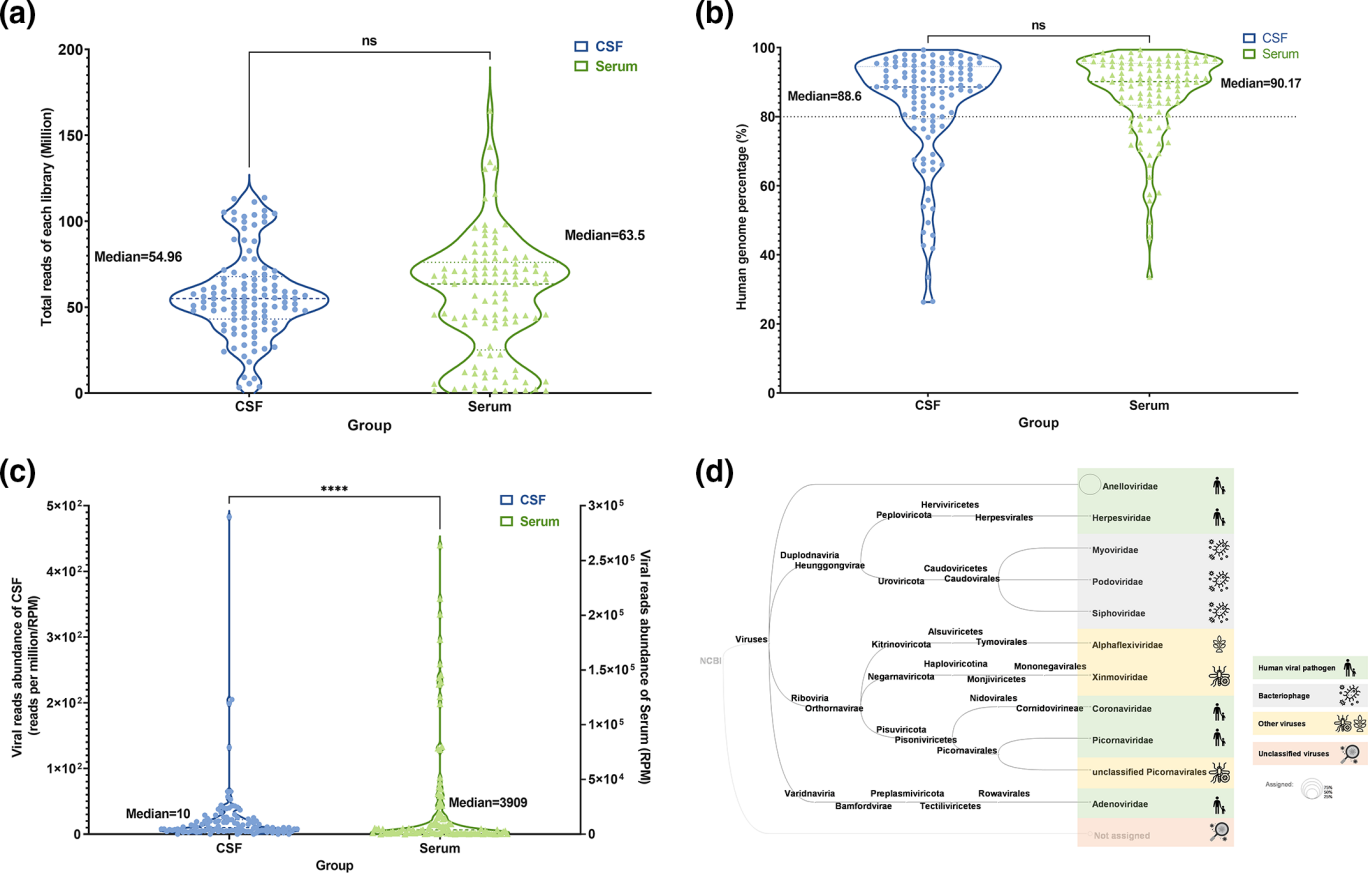
Comparative analysis of CSF and serum samples reveals differences in sequencing and viral detection. (a), (b) and (c) Comparison of the sequencing data volume, host genome percentage, and viral-related reads in CSF and serum samples, respectively. Each point represents a sample, with blue circles indicating CSF samples and green triangles representing serum samples. ns indicates no significant difference (*P*>0.05), and **** indicates a significant difference (*P*<0.05) between the two types of samples. Fig. 2d displays all viruses found in this study, with the corresponding host and the colour block representing the virus classification.

By performing high-throughput sequencing on the samples, we obtained a total of 6,326.1 million paired-end reads from 226 samples. Among them, the median data size of the 113 CSF samples was 54.96 million reads (range: 3.3 to 113.7 million reads), and the median data size of the serum samples was 63.5 million reads (range: 1.2 to 164.2 million reads). There was no significant difference between the two groups (*P*=0.94) (Fig. 2a). The median proportion of the human genome in CSF and serum samples was 88.6 % (range: 26.3–99.4 %) and 90.2 % (range: 33.5–99.4 %), respectively, with no significant difference (*P*=0.11) (Fig. 2b). However, the number of reads annotated as viruses in serum samples was significantly higher than that in CSF samples (*P*<0.0001), with median RPM values of 10 (range: 0 to 483) and 3909 (range: 21 to 263,967), respectively. These results suggesting that serum samples may contain a higher viral load than CSF samples.

Among the 226 samples, we identified a total of 12 viral taxa, including 10 viral families, unclassified Picornavirales, and unclassified virus (reads that could be aligned to viral reference databases but could not be annotated as viruses). Based on the differences in viral hosts, these viral taxa were divided into four categories, including five viral families of human viral pathogens (*Anelloviridae, Herpesviridae, Coronaviridae, Picornaviridae*, and *Adenoviridae*) and three viral families of Bacteriophage (*Myoviridae*, *Podoviridae*, and *Siphoviridae*). In addition, 2 insect-borne viruses (Xinmoviridae and unclassified Picornavirales) and one plant virus (Alphaflexiviridae) were classified as other viruses (Fig. 2d).

To compare the characteristics of cerebrospinal fluid (CSF) and serum samples for viral metagenomic analysis, we further compared the differences in the number and quality of viral contigs obtained from the two sample types. Our results showed that the median number of viral contigs assembled from CSF and serum samples were 6 (range: 0 to 22) and 114 (range: 13 to 2226), respectively, while the median N50 length of viral contigs were 279 bp (range: 172 to 2829 bp) and 784 bp (range: 342 to 2345 bp), respectively. These results indicated that the use of serum samples for viral contig assemble was significantly higher in both quantity and quality than CSF samples (*P* values<0.0001) (Figs. S1. A and B). Additionally, we found that the abundance of viral contigs in both CSF and serum samples was positively correlated with the amount of sequencing data produced (*P* values<0.05) (Fig. S1. C and D).

**Fig. 3.**
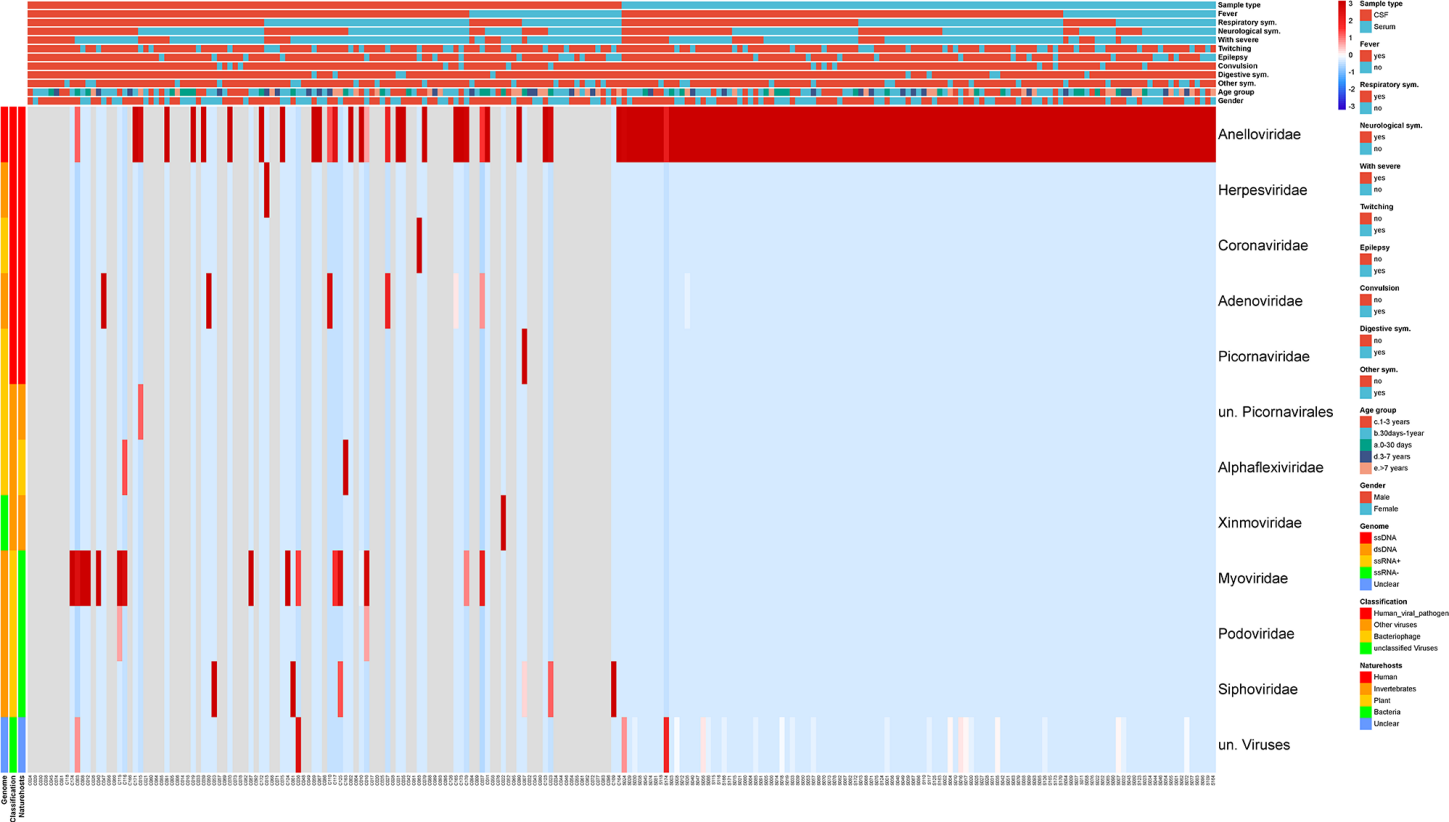
Heat-map revealed the difference in viral profiles between CSF and serum samples. Each column in the figure represents a sample, with CSF on the left and serum on the right. The colour bar on the left displays the classification of viruses, and the colour bar at the top shows the basic characteristics and clinical symptoms of the subjects. The colour of the blocks represents the relative abundance (RPM) of the virus, which has been normalized by each sample for data processing. Red represents high relative abundance, and blue represents low relative abundance.

While the aforementioned results showed that serum samples produced more viral reads or contigs in virome research, our focus is on detecting human viral pathogens for clinical sample diagnosis. Therefore, we analysed the virus profiles identified in CSF and serum samples using a heatmap (Fig. 3). From the heatmap results, we observed that although there was a high relative abundance of viral reads, the variety of viral species present in serum samples was relatively limited, with the viral family of *Anelloviridae* predominating in almost all serum samples. On the other hand, other viral families related to human diseases, including Herpesviridae, Coronaviridae, Adenoviridae, and Picornaviridae, exhibited higher relative abundance in CSF samples. Furthermore, in some CSF samples, viral families related to Bacteriophages also showed higher abundance.

Regarding the overall detection of viruses in each sample type, out of 113 CSF samples, 64 had no detectable viral-related reads, while the remaining 49 samples had a total of more than 95 % relative abundance of Anellovirus, Bacteriophage, and Picornavirus (Figs. S2. A and B). In contrast, Anellovirus was detected in all serum samples and dominated the relative abundance, accounting for 95.6 % of the overall viral profiles. Additionally, for some serum samples, unclassified viruses were also detected, resulting in a total relative abundance of over 99.9 %. The relative abundance of other viruses was extremely low in serum samples (Fig. S2 C. and D). In addition, the quantitative comparison of viral reads in each sample type showed that the number of Anellovirus reads detected in serum (113 positive, median: 3647, range: 10 to 248149) was higher than that in CSF (29 positive, median: 6, range: 2 to 469). On the other hand, the number of Picornaviridae and Adenoviridae reads detected in CSF was higher than that in serum (Table S1).

**Fig. 4.**
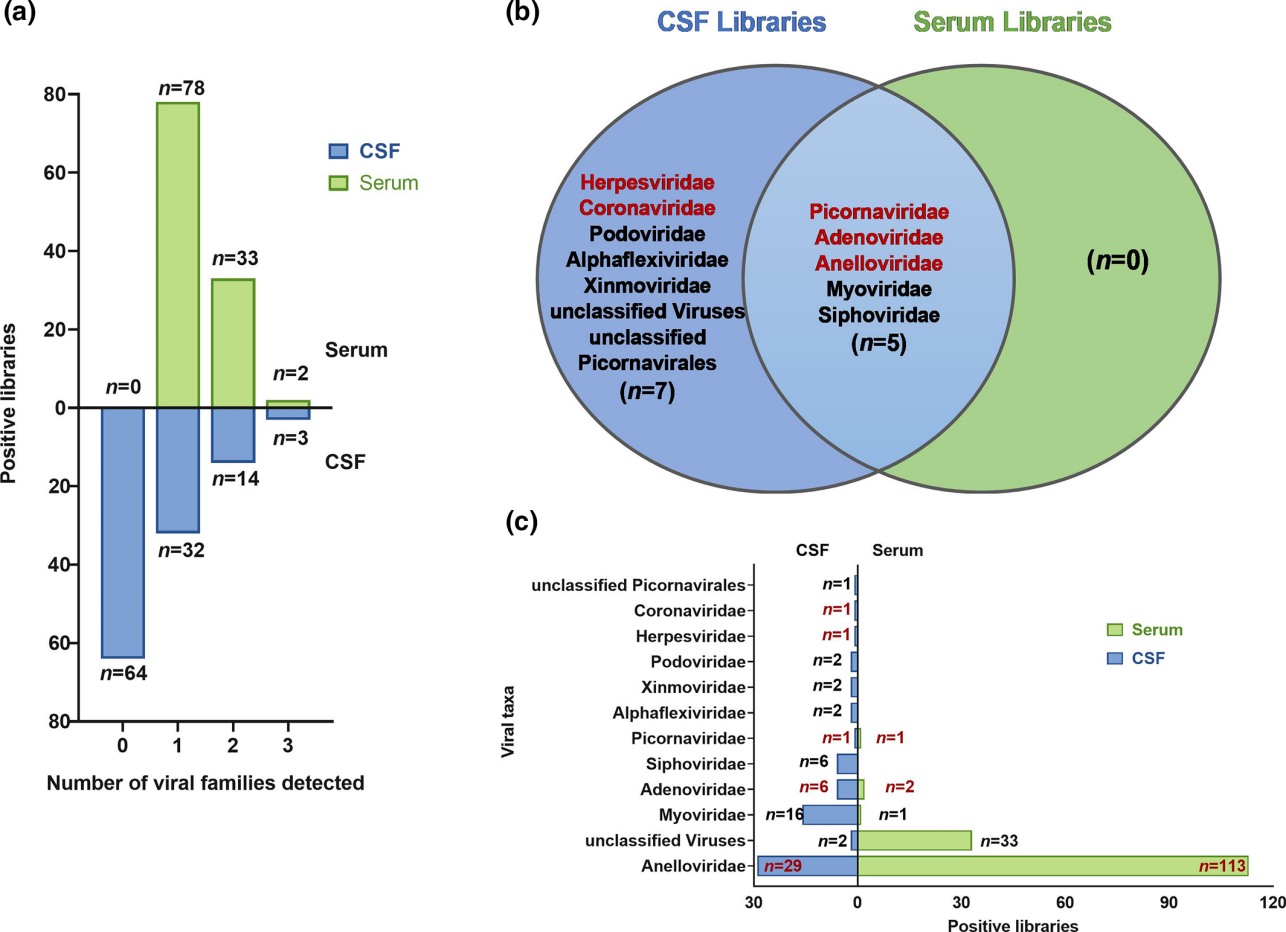
Viruses distribution difference between CSF and serum libraries. In the figure, blue represents CSF libraries and green represents serum libraries. (a) shows the differences in virus detection between CSF and serum. The horizontal axis represents the number of virus types detected in a sample, while the vertical axis represents the number of samples. (b) shows a Venn diagram comparing the viruses detected in the two types of samples. The middle part represents viruses detected in both CSF and serum. (c) shows the number of positive detections of certain viruses in each type of sample.

Regarding the number of viral taxa detected in different samples, one virus was detected in 32 CSF and 78 serum samples, and two or three viruses were detected in 17 CSF and 35 serum samples, suggesting possible cases of mixed infections (Fig. 4a). Venn analysis of the viruses detected in CSF and serum samples revealed the co-existence of five viruses, including three human viruses and two Bacteriophages. Moreover, seven viral taxa were found only in CSF samples (Fig. 4b). Anellovirus was detected in 29 CSF samples and all 113 serum samples, while human viruses of Herpesviridae (one case, 0.9 %) and Coronaviridae (one case, 0.9 %) were detected only in CSF samples. Viral families of Adenoviridae and Picornaviridae were detected in CSF and serum samples, respectively. However, it is worth noting that these viruses detected in both sample types did not originate from the same patient (Fig. 4c and Table 1). In general, although the number and abundance of viruses detected in CSF samples were lower than those in serum samples, the diversity of viruses was higher in CSF samples and more human viral species could be detected. Therefore, CSF samples remain essential for the detection of viral pathogens in patients with meningitis and encephalitis.

**Table 1.** The detection of human viral pathogens in CSF and serum samples

Viruses	Viral taxa	CSF libraries		Serum libraries		Detection in paired CSF and serum
		*N*	Percentage	*N*	Percentage	
Anellovirus	*Anelloviridae*	29	25.7 %	113	100.0 %	29
Herpesvirus	*Herpesviridae*	1	0.9 %	0	0.0 %	0
Coronavirus	*Coronaviridae*	1	0.9 %	0	0.0 %	0
Enterovirus	*Picornaviridae*	1	0.9 %	1	0.9 %	0
Adenovirus	*Adenoviridae*	6	5.3 %	2	1.8 %	0

### Identification and validation of human viral pathogens in patients

Through blast searches on the assembled viral fragments, we were able to identify specific viral types for some of the infected patients (Table 2 and Table S2). To further rule out potential sequencing or analysis errors, we performed PCR amplification and Sanger sequencing validation on the sequences identified by metagenomic analysis as viral. These sequences included those related to herpesvirus, adenovirus and enterovirus detected in CSF or serum. All PCR amplification products were positive, and Sanger sequencing results confirmed a high degree of similarity between these sequences and the corresponding known viruses in the database, further confirming the accuracy of the metagenomic analysis results.

**Table 2.** Classification of viral infections in patients

Subject ID	Sample type	Viral pathogen detected				
		Virus detected	Close strain (Genbank ID/name of reference strain)	Nucleotide sequence identity	Contigs length	Genome covera
subject013	CSF	Herpesvirus (Herpesviridae)	Human herpesvirus 1 strain 17/NC_001806.2	100.0 %	507	0.3 %
subject063	CSF	Coronavirus (Coronaviridae)	Human coronavirus OC43/OK662397.1	98.5 %	686	2.2 %
subject007	CSF	Enterovirus (Picornaviridae)	Echovirus E30/MN153801.1	99.5 %	2271	32.3 %
subject035	Serum	Enterovirus (Picornaviridae)	Coxsackievirus A2/OL519580.1	99.7 %	1105	15.1 %
subject026	Serum	Adenovirus (Adenoviridae)	Human adenovirus B3/MW594185.1	99.6 %	268	0.8 %
subject027	CSF	Adenovirus (Adenoviridae)	Human adenovirus 7 /MW816101.1	96.8 %	332	0.9 %
subject037	CSF	Adenovirus (Adenoviridae)	Human adenovirus 7 /MW816101.1	96.8 %	289	0.8 %
subject047	CSF	Adenovirus (Adenoviridae)	Human adenovirus B3/MW594185.1	100.0 %	276	0.8 %
subject050	CSF	Adenovirus (Adenoviridae)	Human mastadenovirus B/MK847517.1	91.5 %	269	0.8 %
subject086	Serum	Adenovirus (Adenoviridae)	Human mastadenovirus B/MK847517.1	100.0 %	237	0.7 %
subject110	CSF	Adenovirus (Adenoviridae)	Human adenovirus 7 /MW816101.1	96.8 %	252	0.7 %
subject165	CSF	Adenovirus (Adenoviridae)	Human adenovirus 7 /MW816101.1	96.8 %	322	0.9 %
subject011	CSF	Xincheng Mosquito Virus (Xinmoviridae)	Anphevirus *sp*. strain YC899/MW452303.1	79 % (aa:96 %)*	236	1.9 %
subject022	CSF	Xincheng Mosquito Virus (Xinmoviridae)	Anphevirus *sp*. strain YC899 hypothetical protein/MW452303.1	81 % (aa:93 %)	225	1.8 %
subject015	CSF	unclassified Picornavirales	Yongsan picorna-like virus three strain YPLV3/NC_040584.1	100.0 %	790	7.0 %

*aa, amino acid, due to the low identity of nucleotide, close strain was determined based on its amino acid sequence identity.

One severely ill patient had human herpesvirus 1 detected in their CSF, with 100 % nucleotide identity to the reference strain, and exhibited typical neurological symptoms such as fever and twitching. Another patient had human coronavirus OC43 detected in their CSF, with 98.5 % identity to the reference, but only exhibited symptoms of fever, twitching, and epilepsy. In two patients with enterovirus infections, their viruses were detected in both CSF and serum samples. One patient had Echovirus E30 detected in their CSF, with 99.5 % identity to the reference, along with typical neurological symptoms, and was also a severely ill patient. The other patient had Coxsackievirus A2 detected in their serum, with 99.7 % identity to the reference, and exhibited symptoms of only fever and diarrhoea. Of the eight adenovirus infected patients, two were detected in serum samples. Most of these patients exhibited symptoms of fever, respiratory symptoms, and neurological symptoms, but relatively less severe. Although the majority of human viral pathogens were detected in CSF samples (*n*=9), for three cases, viruses related to the diseases were not found in their CSF. However, adenovirus (*n*=2) and enterovirus (*n*=1) were detected in serum samples. This suggests that relying solely on CSF samples for meningitis and encephalitis patients may lead to missing some pathogens, but simultaneous testing of serum samples can increase the likelihood of detecting pathogens to a certain extent.

In addition to the confirmed human viruses, a member of Xinmoviridae (2 cases) and Yongsan picorna-like virus 3 strain YPLV3 (one case, unclassified Picornavirus) were detected in CSF samples. These two viruses are transmitted by mosquitoes and there is no evidence linking them to human diseases. However, all three patients exhibited respiratory symptoms. Among them, both Subject011 and Subject015 have been clinically diagnosed with severe pneumonia (severely ill). Moreover, Subject015 also displays symptoms of fever and twitching (Tables 2 and S2). It is possible that these are incorrectly assign results or co-infections with other pathogens such as bacteria, which warrants further investigation.

## Discussion

Viral infections pose a significant risk to the health and wellbeing of children, and understanding the viral groups that affect patients can aid in diagnosing and treating these illnesses. This study utilized RNA sequencing and virome analysis tools to detect viral pathogens and compare the virome characteristics of paired CSF and serum specimens from hospitalized children. After a rigorous screening process, 113 children were included, and a total of 12 viral taxa were identified in serum and CSF specimens. The *Anelloviridae* family was the most common in serum, while phages were more abundant in CSF. The viral diversity was higher in CSF (12 viral taxa) than in serum (five viral taxa). Among them, one herpesvirus, one coronavirus, one enterovirus, and six adenoviruses were identified in CSF, meanwhile, one anterovirus and two adenoviruses were identified in serum. Our study has revealed the presence of human pathogenic viruses in both CSF and serum specimens in 12 cases where conventional methods failed to determine the cause of encephalitis and meningitis. In general, we utilized mNGS to identify viruses infecting 113 paediatric patients with meningitis and encephalitis. Human-associated viruses were detected in 7.9 %(9/113) of patients using CSF samples, and in 2.6 %(3/113) of patients using serum samples. Simultaneous testing of CSF and serum has the potential to increase the detection rate of pathogens and facilitate the diagnosis of these disease. This finding highlights the possibility that the presence of these viruses in CSF and serum specimens may indicate the aetiology of encephalitis and meningitis, thereby improving the detection rate.

The detection of pathogens in CSF using mNGS is garnering increasing attention. Retrospective studies on confirmed patients have demonstrated that this diagnostic strategy possesses high sensitivity and specificity, whether for viral or bacterial pathogens [26–29]. However, several factors impede the clinical application of CSF. For instance, lumbar puncture is an invasive procedure that can cause pain to patients, and their willingness to undergo such examinations is low. Furthermore, as a cell-free fluid, CSF has low nucleic acid content, restricting the detection of uncertain pathogens. Nonetheless, studies have indicated that mNGS detection through probe enrichment can improve the detection rate of pathogens [30]. In a prospective, multicenter study involving 204 patients (children and adults) with encephalitis and meningitis, 32 pathogens were detected using NGS, of which 19 were viruses (9.3 %, 19/204). Among these viruses, eleven different types such as Coxsackievirus, EBV, and HHV-6 were identified [31]. In our study, we found five viral families of human viral pathogens in 10.6 %(12/113) of patients using both CSF and serum samples. While the results of the two studies cannot be directly compared due to differences in the populations studied, our finding hints at the potential usefulness of serum samples as a complementary diagnostic sample for mNGS-based virus detection. However, NGS detected significantly fewer viral species in CSF and serum compared to other types of human specimens [32]. These results may be attributed to the fact that CSF and serum are cell-free body fluid, and mNGS detects only free nucleic acids in blood. This means that a large number of viruses that are present with cells and bacteria, etc., are not detected.

We have identified virus from the *Coronaviridae* family in a patient, which had 98.45 % nucleotide sequence identity with the human coronavirus OC43. Human coronavirus OC43 is one of four seasonal human coronaviruses that typically cause symptoms of self-limiting common cold. However, some studies have reported that the virus can infect the nervous system and cause fetal encephalitis [33, 34]. Thus, the detection of this virus in CSF samples from hospital children should be treated with great caution. Enteroviruses were found in one CSF and one serum sample. The clinical symptoms of most enterovirus infections are mild flu-like symptoms such as fever, sore throat, headache, and nausea. Nonetheless, in a small number of children, especially those under 5 years of age, enteroviruses may cause severe neurological disorders such as viral encephalitis [35, 36]. Therefore, we believe that enteroviruses found in CSF samples may be the underlying cause of childhood encephalitis. Adenovirus was found both in CSF and serum samples, and its detection rate was higher than other viruses that can cause disease in children. Furthermore, a CSF specimen from a patient with severe encephalitis revealed the presence of human herpesvirus 1 strain (HHV-1), while no other viral species were detected. Therefore, the results can offer further clinical indications for diagnosis and treatment.

Unfortunately, the contigs obtained from our data are relatively short, ranging from 225 to 2271 base pairs, and the genome coverage is relatively low, varying from 0.7–32.3 %. This limitation can be attributed to two factors. Firstly, the nucleic acid yield from CSF or serum samples is generally limited, resulting in lower sample quantities. Secondly, our sequencing data may be insufficient in terms of sequencing depth. Additionally, certain samples contain non-contiguous contigs, as exemplified by Echovirus E30 in subject 007 and Coxsackievirus A2 in subject 035. Furthermore, some contigs are located in non-core regions of the virus, such as the N protein region of human coronavirus OC43 in subject 063. As a result, based on the criteria we have established, we can identify the virus type infecting the subjects using these results. However, the available data is still insufficient to conduct phylogenetic analysis.

Anellovirus were found in 100 % of serum pools and 25.9 % of the CSF pools. The presence of anellovirus is not surprising, as they infect most children before the age of 1 year and establish chronic infections that can be detected in the blood of healthy individuals [37–39]. *Anelloviridae* infections can cause a wide range of clinical manifestations as well as asymptomatic infections in humans. However, currently, there is a paucity of data on these viruses in China. The prevalence of *Anelloviridae* infection is close to 100 % in healthy/eligible populations in countries such as Japan and approximately 10 % in the UK and the USA. While high viral loads of *Anelloviridae* infection have been shown to cause some clinical symptoms in humans, it remains unclear whether they can cause disease.

Contamination presents a serious challenge in mNGS. It can arise from various sources, such as the experimental environment, reagents, cross-contamination during the experiment, and the sampling process. To address this challenge, we implemented several measures in this study. These included the design of blank controls for every experiments run, partitioning of the laboratory environment, and elimination of nucleic acids [10, 12, 14]. The results of our analysis on 64 CSF specimens and five blank controls did not reveal and viral reads, indicating that our procedures effectively controlled contamination from environment, reagents and experimental procedures. Nevertheless, it remains possible that contamination occurred during the sample collection process, given that the specimens were left over from routine testing in the hospital laboratory. Moreover, when analysing the results, we applied a set of criteria to eliminate potentially false-positive outcomes that could have resulted from contamination or mismatches. These criteria primarily comprised of: (1) a minimum of 2 RPM of reads provisionally assigned to the viral genome, (2) the read abundance being represented by more than 1 % of the total number of viral reads, and (3) at least one assembled fragment that accurately aligned with the corresponding virus. By introducing these judgement criteria, it is evident that the accuracy of the analysis results is significantly improved [40].

Both CSF and serum specimens are cell-free body fluids in humans, and therefore both of them have low levels of nucleic acids. As a result, enrichment of pathogens is a crucial additive strategy used in mNGS that can significantly enhance the sensitivity of the assay [41]. Enrichment primarily involves two strategies: the first involves the indirect enrichment of pathogen nucleic acids by removing host genomic DNA and rRNA [42]. This method is not appropriate for specimens with low nucleic acid content such as serum and CSF specimens without cellular body fluids, as it may result in insufficient nucleic acid library construction and loading volume. The second method is direct enrichment of nucleic acids using probe, which may introduce bias in virome compared to random amplification of MDA detection process [43, 44]. Thus, we opted not to use enrichment methods for pathogens during our study.

Our study has some limitations. It was conducted solely on hospitalized children, and lacked controls of healthy children to compare the viral characteristics in CSF and serum between healthy and hospitalized children. Additional, the blood–brain barrier presents inherent differences in the distribution of viruses that cause encephalitis in CSF and serum. These factors may limit the ability of CSF and serum samples to provide ideal representations of disease, and future studies should include more diverse disease samples. This study only focused on CSF and serum, as they are the most commonly used samples in clinical practice. However, other types of samples should be prioritized in future studies. Furthermore, as a majority proportion of the study participants in this research are infants or children, obtaining accurate onset times of symptoms through patient self-reporting is uncertain. Therefore, it is challenging to determine whether the sample time falls within the acute phase, which may affect the detection of viruses in cerebrospinal fluid and serum samples. In future studies, researchers should take into account the potential impact of the duration between onset and sampling on pathogen detection. Moreover, nucleic acid detection methods, including PCR and sequencing, are powerful tools for identifying viral sequences in samples. However, the presence of viral nucleic acids does not necessarily indicate active viral replication or the direct role of the detected virus in causing the observed clinical symptoms. Thus, it is important to note that nucleic acid detection alone does not provide concrete evidence of viral infectivity or causation of infection and symptoms. Integration of these methods with other approaches, including viral culture, serology and epidemiology studies, is crucial for establishing a comprehensive understanding of viral infections and their role in clinical manifestations.

## Conclusion

The results suggest that there are distinct viral profiles between the CSF and serum samples, with CSF samples exhibiting a higher proportion of human viral pathogens compared to serum samples. However, the combined use of these two types of samples can increase the detection rate of human viral pathogens in children with encephalitis and meningitis. Additionally, the results provide insight into the diversity of viruses that may contribute to these diseases, which can inform improved diagnostic and therapeutic strategies. However, further investigations are required to validate these findings and optimize the utilization of different sample types in virome analysis for clinical purposes.

## Supplementary Data

Supplementary material 1
