# Supplementary material for: RNA-sequencing-based detection of human viral pathogens in cerebrospinal fluid and serum samples from children with meningitis and encephalitis
Source: Microb Genom. 2023 Aug 2;9(8):mgen001079. doi: 10.1099/mgen.0.001079 (PMC10483426; doi:10.1099/mgen.0.001079)
Supplement: Supplementary material 1 [file mgen-9-1079-s001.pdf]

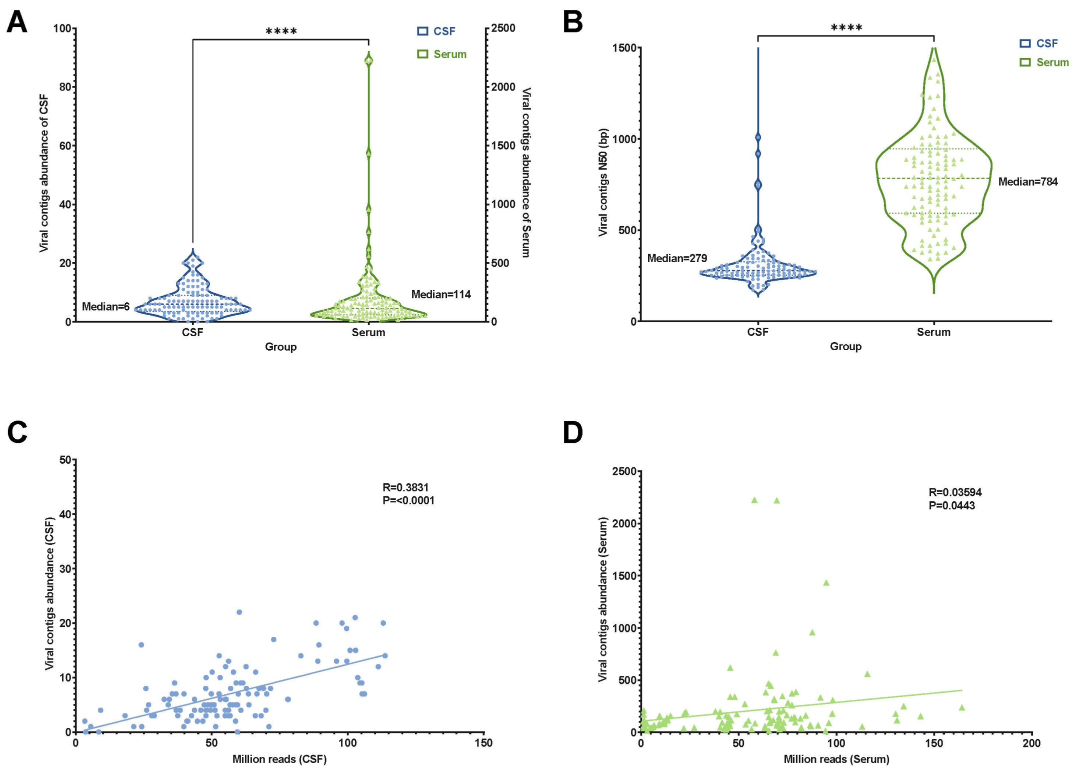

Figure.S1 Comparison of Viral Contigs Assembly Performance between CSF and Serum Samples. A and B, the number and length (N50) of viral contigs in CSF and Serum samples were compared. Blue circles indicate one CSF sample, green triangles indicate one Serum sample, and **** indicates a significant difference ($P<0.05$) between the two groups. C and D show the correlation analysis between the number of viral contigs and the sequencing data volume in the two types of samples.

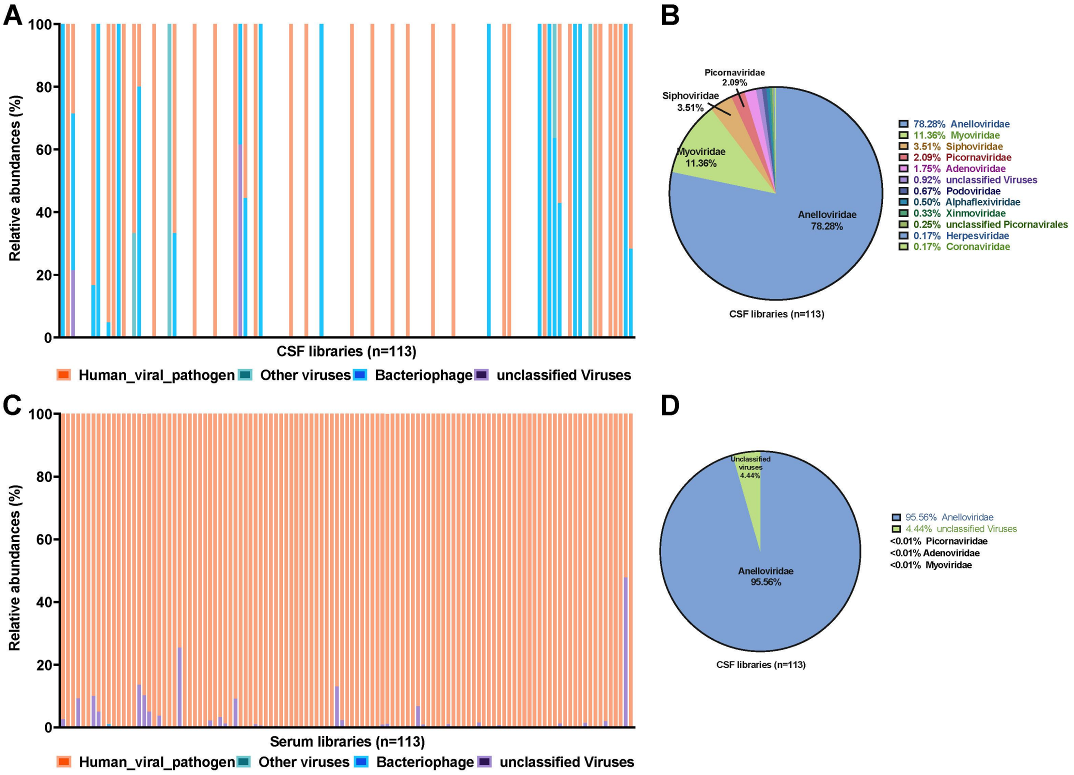

Figure.S2 The Relative Abundance of Viruses in CSF and Serum Samples. A and B show the percentage distribution of viruses in each CSF sample as well as the overall distribution in 113 CSF samples. C and D show the percentage distribution of viruses in individual samples and the overall distribution in 113 CSF samples.

Table.S1 Number of Viral Reads Detected in CSF and Serum Libraries.

Classification	Viral Taxa	Total positive subjects	Total positive libraries	Detection in paired CSF and Serum	CSF libraries					Serum libraries				
					n	Percentage	Min RPM	Max RPM	Median RPM	n	Percentage	Min RPM	Max RPM	Median RPM
Human_viral_pathogen	Anelloviridae	113	142	29	29	25.7%	2	469	6	113	100.0%	10	248149	3647
Human_viral_pathogen	Herpesviridae	1	1	0	1	0.9%	2	2	2	0	0	-	-	-
Human_viral_pathogen	Coronaviridae	1	1	0	1	0.9%	2	2	2	0	0	-	-	-
Human_viral_pathogen	Picornaviridae	2	2	0	1	0.9%	25	25	25	1	0.9%	6	6	6
Human_viral_pathogen	Adenoviridae	8	8	0	6	5.3%	2	9	2.5	2	1.8%	5	6	5.5
Other viruses	unclassified Picornavirales	1	1	0	1	0.9%	3	3	3	0	0	-	-	-
Other viruses	Xinmoviridae	2	2	0	2	1.8%	2	2	2	0	0	-	-	-
Other viruses	Alphaflexiviridae	2	2	0	2	1.8%	2	4	3	0	0	-	-	-
Bacteriophage	Myoviridae	16	17	1	16	14.2%	2	25	6.5	1	0.9%	2	2	2
Bacteriophage	Podoviridae	2	2	0	2	1.8%	3	5	4	0	0	-	-	-
Bacteriophage	Siphoviridae	6	6	0	6	5.3%	2	17	5.5	0	0	-	-	-
unclassified Viruses	Not assigned	35	35	0	2	1.8%	3	8	5.5	33	29.2%	3	96068	138

Table.S2 Clinical Symptoms of Patients.

SubjectID	Characteristics		Sample type	Clinical symptoms								
	Gender	Age		Days of Fever	Respiratory symptoms	Neurological symptoms	Patients with severe	Twitching	Epilepsy	Convulsion	Digestive system symptoms	Other symptoms
subject013	Male	4y8m	CSF	4 days	no	yes	yes	yes	no	no	no	no
subject063	Male	1y8d	CSF	21 days	no	no	no	yes	yes	no	no	no
subject007	Male	3d	CSF	no fever	no	yes	yes	no	no	no	no	no
subject035	Male	1y3m	Serum	4 days	no	no	no	no	no	no	yes	no
subject026	Male	9y9m	Serum	13 days	yes	yes	no	no	no	no	no	yes (Headache)
subject027	Male	5m10d	CSF	NA*	no	no	no	yes	no	no	no	no
subject037	Male	20d	CSF	no fever	yes	yes	no	yes	no	no	no	no
subject047	Female	2m14d	CSF	1 day	yes	yes	no	no	no	no	no	no
subject050	Female	8m17d	CSF	5 days	yes	no	no	no	no	no	no	no
subject086	Female	4m1d	Serum	9 days	no	yes	no	no	no	no	no	no
subject110	Male	3y7m	CSF	6 days	no	yes	no	no	no	no	no	no
subject165	Female	7m	CSF	6 days	no	no	no	no	no	no	no	no
subject011	Male	4d	CSF	no fever	yes	no	yes	no	no	no	no	no
subject022	Male	15d	CSF	no fever	yes	no	no	yes	no	no	no	no
subject015	Male	10y9m	CSF	NA	yes	no	yes	yes	no	no	no	no

*NA,Not Available
